# The EORTC updated breast cancer quality of life questionnaire EORTC QLQ-BR42: A psychometric study with Spanish patients

**DOI:** 10.1186/s12885-026-15831-8

**Published:** 2026-03-14

**Authors:** Juan Ignacio Arraras, Jose Juan Illarramendi, Ana Manterola, Johannes M Giesinger, Susana de la Cruz, Lucia Teijeira, Angels Pont, Vesna Bjelic-Radisic, Esteban Salgado, Maria Jose Lecumberri, Angela Fernandez de Lascoiti, Ruth Vera

**Affiliations:** 1 Hospital Universitario de Navarra: Medical Oncology Department, Pamplona, Spain; 2 Hospital Universitario de Navarra: Radiotherapeutic Oncology Department, Pamplona, Spain; 3 Navarra Institute for Health Research (IdiSNA), Pamplona, Spain; 4 University Hospital of Psychiatry II, Medical University of Innsbruck, Innsbruck, Austria; 5 Health Services Research Group, IMIM (Hospital del Mar Medical Research Institute), Barcelona, Spain; 6 CIBER in Epidemiology and Public Health, CIBERESP, Barcelona, Spain; 7 Helios University Clinic: Breast Unit, Wuppertal, Germany; 8 University Witten/Herdecke, Witten, Germany

**Keywords:** Breast cancer, Quality of life, Spanish, Questionnaire, Psychometrics, EORTC

## Abstract

**Background:**

The EORTC Quality of Life Group recently developed the new version of its breast-cancer-specific quality of life questionnaire, the EORTC QLQ-BR42. This questionnaire updates EORTC QLQ-BR23, which was published in 1996. Since then, significant changes have occurred in breast cancer treatment. The aims of this study were to evaluate the psychometric properties of the QLQ-BR42 in Spanish breast cancer patients and compare the results with those of the EORTC international validation study.

**Methods:**

A retrospective analysis of QLQ-BR42 data from three Spanish breast cancer patient cohorts was conducted to evaluate the questionnaire’s psychometric properties, including scale structure, internal consistency, test-retest reliability, validity (convergent, discriminant, known-groups), and responsiveness to change. The three cohorts comprised, respectively, patients in stages I-III undergoing endocrine treatment, patients with metastatic disease, and patients in stages I-III diagnosed with COVID-19. The metastatic and COVID-19 cohorts completed the EORTC QLQ-BR42 and the EORTC QLQ-C30 once each, while the endocrine treatment cohort completed the questionnaires three times.

**Results:**

A total of 516 patients were included in the study (*N* = 159 in the endocrine treatment cohort, *N* = 98 in the metastatic patients cohort and *N* = 259 in the COVID-19 cohort). Confirmatory factor analyses generally supported the structure of all scales included (RMSEA = 0.051).

Factor loadings were above 0.60, except for the systemic chemotherapy side effects (SCSE) scale, which includes three items with factor loadings ranging from 0.29 to 0.41.

Correlations with (un)related QLQ-C30 scales supported convergent validity (*r* > 0.40) and discriminant validity (*r* < 0.10). The QLQ-BR42 scales distinguished between stage I-III patients who received endocrine treatment and those with metastatic disease, as well as between surgical approaches. Substantial changes over time were observed in patients whose global QOL (measured with QLQ-C30 item #30) had improved or worsened.

**Conclusions:**

The EORTC QLQ-BR42 is a reliable and valid tool when used with a sample of Spanish breast cancer patients, although the SCSE requires further research on its internal consistency. These findings generally match those of the EORTC international validation study.

**Supplementary Information:**

The online version contains supplementary material available at 10.1186/s12885-026-15831-8.

## Background

With an incidence in 2024 of 36,395 new cases, breast cancer (BC) is the most prevalent type of cancer among women in Spain [1]. The gradual increase in incidence – associated with an ageing population, the implementation of screening programmes, and declining mortality rates – has led to a growing number of breast cancer survivors (BCSs) [2]. In Spain, BC survival at 5 years is over 80% [1]. However, although some cancer-related concerns tend to decrease with time, breast cancer treatments can leave physical, psychological and psychosocial sequelae that may interfere with their Quality of Life (QOL) [3].

The EORTC (European Organisation for Research and Treatment of Cancer) Quality of Life Group (QLG) developed a modular system for QOL assessment that comprises a general core questionnaire, the QLQ-C30 [4], plus complementary modules for different tumour sites or other areas, such as fatigue. The EORTC breast cancer module QLQ-BR23 [5] was one of the first QOL questionnaire modules developed for use in conjunction with the EORTC QLQ-C30. Our group validated QLQ-C30 and QLQ-BR23 for use in Spain [6, 7].

Since the QLQ-BR23 was created, new therapeutic options for BC have been added, including targeted therapy (TT) and immunotherapy agents [8–10], while treatments such as surgical techniques, endocrine therapy (ET) and radiotherapy (RT) have been improved [11, 12]. The EORTC QLG therefore decided to update the QLQ-BR23 module since it considered that the original QLQ-BR23 could not cover many important QOL issues or potential side effects [13].

An updated version, the QLQ-BR42, has recently been created and validated in an international sample [14]. This updated version (Supplementary Table 1) comprises the 23 items of the QLQ-BR23 plus 19 items covering new areas (hand/foot symptoms/neuropathy, skeletal symptoms, vaginal symptoms, breast satisfaction and weight gain). Authors who have conducted QOL studies of ET in BC patients recommend administering this updated version of the EORTC breast cancer questionnaire since it captures the impact of ET better than the QLQ-B23 [15].

The EORTC QLG recommends that studies be conducted in individual countries to provide deeper insight into the functioning of the QOL instruments in each national context [4]. However, no psychometric study of the QLQ-BR42 has yet been published in any individual country.

The aims of this study are to evaluate the structure, reliability and validity of the QLQ-BR42 questionnaire when applied to a sample of Spanish BC patients and compare the results of these analyses with those of the validation study conducted by the EORTC QLG [14].

## Methods

### Study samples

BC patients from three prospective cohorts treated at the Hospital Universitario de Navarra (Spain) were included in this study. Combining these cohorts enabled us to perform more solid analyses. The common inclusion criteria for all three cohorts were: being 18 or older; having been diagnosed with primary or recurrent BC; and understanding and able to complete the questionnaire in the language in which it was administered. Patients with concurrent malignancies were excluded.

The three cohorts were:

1. Postmenopausal patients diagnosed with stage I-III BC who had received ET for five years following surgery and who had or had not received chemotherapy (CT) and/or RT. Patients completed the QOL questionnaires: during the final week of their five-year ET course; six months later; and one year later [16].
2. Patients with stage IV BC diagnosed at least 48 months previously, regardless of whether the diagnosis was de novo or a relapse. Since their diagnosis of metastatic disease, the patients may have undergone one or more lines of treatment. Participants included those receiving or having received ET, CT, TT and/or surgical interventions. These patients, and those from the following sample, completed the questionnaires once [17].
3. Patients diagnosed with stage I-III BC and COVID-19 who had undergone surgery and received any cancer treatment (CT, RT and/or ET) or who were in follow‐up between February and September 2021 [18]. We included this cohort as we wanted to check whether the psychometric properties of the QLQ-BR42 are also good if the questionnaire is used both in breast cancer patients with a specific comorbidity and in the unusual context of the pandemic. This enabled us to assess the robustness of these psychometric properties.

### Measures

All patients completed EORTC QOL questionnaires QLQ-C30 (Quality of Life core questionnaire) [3] and QLQ-BR42 [14]. Questionnaires with under 70% of items answered were excluded. Table S1 in the supplementary material shows the main features of these questionnaires. QLQ-C30 evaluates areas common to various tumour sites and their associated treatments. The two EORTC questionnaires were translated into Spanish in accordance with EORTC translation procedure [19].

The treating oncologist evaluated limiting comorbidity using the Charlson questionnaire [20] and assessed performance status using the Karnofsky scale [21]. These assessments were conducted at longer sessions on days scheduled for habitual consultations in the oncology departments. QOL assessments were also conducted in person during these sessions (rather than by telephone, mail or email) to avoid burdening the patients with additional visits and decrease the risk of missing data. Other demographic and clinical data were collected by physicians from clinical records or patient interviews.

The three studies (project numbers 2018/64, PI_2020/106 and 2019/57, respectively) followed the recommendations of the Declaration of Helsinki and were approved by the Regional Ethics Committee for Biomedical Research of Navarra, Spain. All patients signed written informed consent forms.

### Statistical analysis

Descriptive analyses were conducted on the sociodemographic and clinical data (number and percentages). Data from all patients were used to evaluate the scale structure, internal consistency, and convergent and discriminant validity.

Confirmatory factor analysis was performed to confirm the hypothesised scale structure based on data from the international study [14]. Data from the three samples were combined to obtain more heterogeneity in the distribution and a sample size large enough to perform individual factor analysis. Confirmatory factor analysis (CFA) was conducted using the WLSMV (weighted least squares mean and variance adjusted) estimator. In this analysis, standardised factor loadings for each item on the corresponding scale were expected to be > 0.60 [22, 23]. Items were dichotomised due to low frequency in some response categories to ensure sufficient cell counts for the analysis. Scores for each item were dichotomised between the response with the highest QOL and the other three options. Model fit was evaluated using standard fit indices, and standardised factor loadings were examined. Goodness-of-fit indices indicated good model fit if values were above 0.95 for the Comparative Fit Index (CFI) and the Tucker-Lewis index (TLI), and below 0.05 for the Root Mean Square Error of Approximation (RMSEA) [24]. A path diagram illustrating the latent and observed variables was generated using the semPaths function.

Floor and ceiling effects were analysed for all scales: >15% response at the extremes indicated these effects [25, 26]. Two types of reliability were assessed, i.e. internal consistency and test-retest (see below). Internal consistency was measured using Cronbach’s alpha, with values > 0.70 considered ‘acceptable’ and those > 0.80 considered ‘good’ [27]. Item 30 of the EORTC QLQ-C30 (on general QOL) was used to assess stability (test-retest reliability) as well as deterioration or improvement (responsiveness-to-change).

Test-retest reliability of the QLQ-BR42 was assessed in the ET cohort among patients whose health status remained stable, defined as no change in item 30 of the QLQ-C30 between the second and third assessments (six months apart). The Intraclass Correlation Coefficient (ICC) was calculated and no significant changes were expected [28].

Convergent and discriminant validity were assessed using Spearman’s rank correlation, with convergent validity indicated by the item-to-scale (corrected for overlap) correlations ≥ 0.40 and discriminant validity indicated by correlations ≤ 0.50 with other scales [29, 30]. Convergent and discriminant validity were also assessed using Spearman’s rank correlation: the relationship between the scales of the QLQ-BR42 and the QLQ-C30 were studied, with > 0.40 and < 0.30 indicating sufficient convergent [31, 32] and discriminant validity [32], respectively. Higher correlations were expected among areas whose content may be more related: e.g., physical functioning (QLQ-C30) and the skeletal scale; the pain scale (QLQ-C30) and the skeletal scale. Lower correlations were expected among areas whose content may be less related: e.g., constipation (QLQ-C30) and future perspective; diarrhoea (QLQ-C30) and breast symptoms.

Known-groups validity was evaluated by comparing QLQ-BR42 scales and items using Student’s t-tests of the differences between the cohorts. Comparisons were first conducted between the ET and metastatic disease cohorts. Then, within the ET cohort, sub-groups based on the type of breast surgery (conservative or radical) were compared. Higher QOL scores on the QLQ-BR42 were expected in patients from the ET cohort than in patients from the metastatic disease cohort [33] and in patients who had undergone conservative surgery than in those who had undergone radical surgery [34].

Responsiveness to change in the QLQ-BR42 was assessed in the ET cohort by dividing patients into two sub-groups based on changes in item 30 of the QLQ-C30 (general QOL) between the first and second assessments six months apart. The first sub-group comprised patients whose QOL improved by *≥* 1 point, and the second comprised patients whose QOL worsened by *≥* 1 point. Paired t-tests were used to evaluate differences between the two assessments. In the first analyses, significant QOL improvements were expected in functioning (e.g., future perspective) and symptoms (e.g., skeletal) areas, whereas in the second analyses significant worsening of QOL was expected in functioning (e.g., breast satisfaction) and symptoms (e.g., hand/foot symptoms and neuropathy). All analyses were conducted using the R software package [35].

## Results

A total of 516 patients were included in this study (159 in the stage I-III cohort of patients who had received ET; 98 in the metastatic cohort; and 259 in the BC/COVID-19 cohort). The mean age for all patients was 65.1. Various cohabitation and oncology treatment modalities were represented (Table 1).

**Table 1:** Descriptive characteristics of the global sample and the three study groups

	All	ET cohort	Metastasis cohort	Covid cohort	*p*-value
Sociodemographic					
Age, mean (SD)	65.1 (10.0)	69.2 (9.3)	65.1 (10.0)	62.6 (9.6)	< 0.001
Marital status, *n* (%)					
Single	53 (10.4%)	26 (16.6%)	3 (3.1%)	24 (9.4%)	< 0.001
Married	381 (74.7%)	106 (67.5%)	80 (82.5%)	195 (76.2%)	
Separated	30 (5.9%)	3 (1.9%)	3 (3.1%)	24 (9.4%)	
Widowed	46 (9.0%)	22 (14.0%)	11 (11.3%)	13 (5.1%)	
Cohabitation, *n* (%)					
Alone	65 (13.1%)	31 (19.7%)	13 (13.4%)	21 (8.7%)	< 0.001
With a partner	284 (57.3%)	103 (65.6%)	56 (57.8%)	101 (51.6%)	
With children over 18	120 (24.2%)	8 (5.1%)	24 (24.7%)	88 (36.4%)	
With other people	27 (5.40%)	15 (9.6%)	4 (4.1%)	8 (3.3%)	
CLINICAL					
Karnofsky, mean (SD),	80.0 (8.9)	83.9 (8.7)	74.5 (11.2)	79.5 (6.9)	< 0.001
range	50–100	60–100	50–100	60–90	
Karnofsky 3 categories, *n* (%)					
90–100	144 (29.0%)	78 (50.6%)	13 (15.7%)	53 (20.4%)	< 0.001
80	223 (44.9%)	50 (32.5%)	29 (34.9%)	144 (55.4%)	
50–70	130 (26.2%)	26 (16.9%)	41 (49.4%)	63 (24.2%)	
Breast surgery, *n* (%)					
conservative	258 (51.4%)	111 (71.6%)	56 (60.9%)	91 (35.7%)	< 0.001
radical	244 (48.6%)	44 (28.4%)	36 (39.1%)	164 (64.3%)	
Axillary surgery, *n* (%)					
Axillary node dissection	107 (21.9%)	36 (23.2%)	35 (43.2%)	36 (14.2%)	< 0.001
Sentinel node biopsy	369 (75.5%)	115 (74.2%)	45 (55.6%)	209 (82.6%)	
No	13 (2.7%)	4 (2.6%)	1 (1.2%)	8 (3.2%)	
TREATMENT					
Hormonotherapy					
No	75 (15.0%)	0 (0.0%)	24 (27.6%)	51 (19.8%)	a: < 0.001 b: < 0.001 c: 0.126
Yes	424 (85.0%)	154 (100.0%)	63 (72.4%)	207 (80.2%)	
Chemotherapy *					
No	403 (80.8%)	95 (61.3%)	77 (91.7%)	231 (88.8%)	a: < 0.001 b: < 0.001 c: 0.463
Yes	96 (19.2%)	60 (38.7%)	7 (8.3%)	29 (11.2%)	
Radiotherapy *					
No	77 (0.9%)	17 (11.0%)	30 (36.1%)	47 (18.1%)	a: < 0.001 b: 0.003 c: 0.001
Yes	266 (0.1%)	137 (89.0%)	53 (63.9%)	213 (81.9%)	

In the ET cohort the frequencies for chemotherapy and radiotherapy refer to previous treatments, while in the Metastasis and Covid cohorts frequencies refer to current treatment

*P*-values: a: comparing all cohorts; b: comparing ET cohort vs. Metastases and Covid cohorts; c: comparing Metastases vs. Covid cohort

*ET cohort past treatment: chemotherapy and radiotherapy

Of the QLQ-BR42 items, 26 had complete responses with no missing data. For nine items, the number of patients who failed to respond ranged from 1 to 5 (0.96% of all patients). Item 68, which assesses discomfort in the vagina, had 85 missing responses (16.5% of patients). Items 44 (interest in sex) and 45 (sexual activity) were missing for 206 and 226 patients, respectively. Certain items had missing data due to their conditional nature: item 35 (upset by hair loss) was missing for 373 patients as it only applies to those who experienced hair loss (135 patients); item 46 (sexual enjoyment) was missing for 344 patients and items 69 and 70 (pain and vaginal dryness) were missing for 307 patients each (Supplementary Table 2). These last three items apply only to patients who were sexually active (155 patients reported engaging in some sexual activity in item 45).

Patients who responded to the two sexual functioning items had a higher performance status and were younger, more likely to be married or living with a partner, and more likely to be receiving or to have received radiotherapy (in the past or present) than those who omitted a response on the Sexual Functioning scale (Supplementary Table 3).

### Factor analyses

Some items were not included in the analyses. As in the international study [14], the items on vaginal symptoms (69 and 70) were not included due to the high number of missing answers. Item 68 was not included as it was intended to be a component of the vaginal symptoms scale. Item 45 was not included as no patient selected the highest score, making dichotomisation impossible. Item 44 was also excluded, as it is intended to form a two-item sexual functioning scale together with item 45. Moreover, items 44 and 45 had a high number of missing items. Item 35 had few answers and was also excluded. Item 43 (worries about future health) and items 46 and 67 (weight gain) were excluded from the analyses because they are individual items. Item 40 (feeling less feminine, from the body image scale) and item 71 (satisfaction with the cosmetic result of surgery, from the breast satisfaction scale) also did not fit the model and were excluded from the final version. Item 72 (satisfaction with the appearance of the skin of the affected breast), which was intended to form a two-scale item with item 71, was also excluded from the model. The items included in and excluded from the model, and the reasons for not including the excluded items are presented in Supplementary Table 2.

**Fig. 1:**
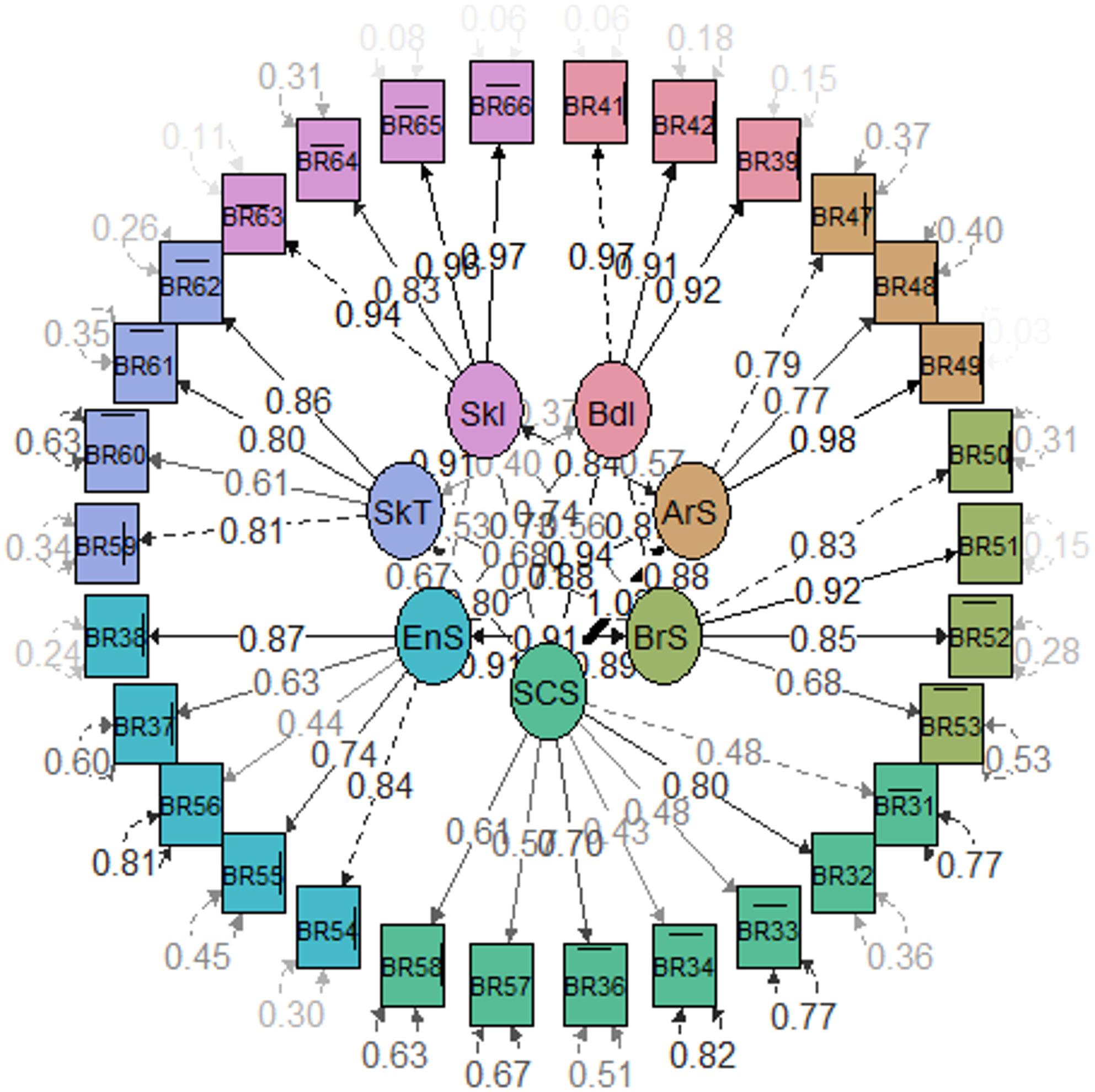
Path diagram. Goodness of fit measures: confirmatory factor analysis of the items included in the analyses. CFI = 0.952, TLI = 0.946, RMSEA = 0.051 (95%CI: 0.046–0.056) BR31 to BR66: number of the QLQ-BR42 items. QLQ-BR42 scales: BdI, Body image; ArS, Arm symptoms; BrS, Breast symptoms; SCS, Systemic chemotherapy side effects; EnS, Endocrine symptoms; SkT, Hand/foot symptoms/neuropathy; SkL, Skeletal symptoms

The final structure showed that most standardised factor loadings ranged from 0.60 to 0.99, indicating adequate representation of items for their respective latent constructs (Fig. 1). Exceptions were observed in the systemic chemotherapy side effects domain, where three items (31, 33, and 34) exhibited poor loadings (0.29–0.41) (Supplementary Table 4). Goodness-of-fit indices from the confirmatory factor analysis of the included items confirmed the hypothesised scale structure of the questionnaire (CFI = 0.952, TLI = 0.946, RMSEA = 0.051).

### Frequencies

Frequencies for the scales and individual items from the first QLQ-BR42 assessment are presented in Table 2. All areas ranged from 0 to 100, except for sexual functioning, systemic chemotherapy side effects, endocrine symptoms, and hand/foot symptoms/neuropathy. Floor effects were found in the sexual functioning, sexual enjoyment, arm symptoms, breast symptoms, vaginal symptoms, endocrine symptoms, hand/foot symptoms/neuropathy, and weight gain scales (16.9%–70.3%). Ceiling effects were found in the body image, future perspective and breast satisfaction scales (36.9%–74.4%).

**Table 2:** Examination of the distribution of dimension scores. *Distribution of dimension scores. Multi-item scales *(*n = 516)*

	**Body Image**	**Sexual Function**	**Sexual Enjoyment**	**Future Perspective**	**Breast Satisfaction**	**Arm Symptoms**	**Breast Symptoms**
Theoretical range	0–100 (Worst – Best)	0–100 (Worst – Best)	0–100 (Worst – Best)	0–100 (Worst – Best)	0–100 (Worst – Best)	0–100 (Best – Worst)	0–100 (Best – Worst)
Number of items	4	2	1	1	2	3	4
Observed range	0–100	0–66.7	0–100	0–100	0–100	0–100	0–66.7
Mean (SD)	92.7 (17.0)	19.2 (19.0)	37.8 (22.8)	70.4 (30.7)	73.4 (25.9)	13.7 (20.1)	7.6 (13.3)
Missing, n (%)	0 (0.0%)	226 (43.8%)	344 (66.7%)	3 (0.6%)	1 (0.2%)	5 (1.0%)	0 (0.0%)
Floor effect	1.0%	43.4%	16.9%	5.5%	3.7%	51.7%	61.4%
Ceiling effect	74.4%	0.0%	0.0%	43.1%	36.9%	1.0%	0.0%
Cronbach’s Alpha Original Scale	0.88	0.73	--	---	0.95	0.75	0.71
	**Systemic Chemo Side Effects**	**Vaginal Symptoms**	**Endocrine Symptoms**	**Hand/foot symptoms/neuropathy**		**Weight Gain**	**Skeletal**
Theoretical range	0–100 (Best – Worst)	0–100 (Best – Worst)	0–100 (Best – Worst)	0–100 (Best – Worst)		0–100(Best – Worst)	0–100 (Best – Worst)
Number of items	8	3	5	4		1	4
Observed range	0–70.8	0–100	0–86.7	0–75		0–100	0–100
Mean (SD)	15.9 (13.4)	9.2 (19.6)	11.7 (15.8)	19.8 (20.3)		23.3 (28.3)	37.0 (27.4)
Missing, n (%)	5 (0.9%)	307 (59.5%)	3 (0.6%)	0 (0%)		0 (0%)	0 (0%)
Floor effect	0.0%	70.3%	40.5%	28.9%		50.7%	2.5%
Ceiling effect	10.9%	2.0%	0.0%	0.0%		4.9%	15.7%
Cronbach’s Alpha Original Scale	0.55	0.91	0.70	0.73		---	0.89

### Reliability

Cronbach’s alpha coefficients exceeded 0.70 for most scales and were above 0.80 in four cases. The systemic chemotherapy side effects scale showed a lower alpha (0.55), reflecting its limited score range (0–70.8) and the predominance of responses in the lower categories. For all items on this scale (except item 35, for which 69.5% of responses were in the two lowest categories), over 75% of responses fell into these “not at all” or “a little” categories. Five items – including item 32, for which 89.1% of responses were “not at all” – had more than 93% of responses in these categories.

### Test-retest

ICCs between the second and third assessments for patients whose status remained stable (no change in item 30) were *moderate* (0.5–0.75 for three QLQ-BR42 scales; *good* (0.75–0.90) for four QLQ-BR42 scales, and *excellent* (> 0.90) for six scales [36] (Supplementary Table 5).

### Convergent and discriminant validity

Item-to-scale correlations were ≥ 0.40 for most items. Exceptions were items 41 (problems looking at oneself naked) and 56 (being dizzy), which were close to the threshold (0.36 and 0.35), as well as items 32–36, 57 and 58 from the systemic chemotherapy side effects scale. All these items also showed low discriminant correlations, with the highest value being 0.43. Additionally, all QLQ-BR42 items except item 59, which was very close to the threshold (0.52), had discriminant correlations < 0.50 (Supplementary Table 6).

Overall, the scales of the QLQ-C30 and QLQ-BR42 did not exhibit high correlations. The highest correlations (> 0.4) were found between: physical functioning (QLQ-C30) and the systemic chemotherapy side effects (-0.46) and skeletal (-0.50) scales; global QOL (QLQ-C30) and the systemic chemotherapy side effects (-0.42) and skeletal (-0.42) scales; fatigue (QLQ-C30) and the future perspective (-0.42), vaginal symptoms (0.53), systemic chemotherapy side effects (0.41) and skeletal (0.51) scales; pain (QLQ-C30) and the hand/foot symptoms/neuropathy (0.40) and skeletal (0.57) scales. Very low correlations (< 0.1) were found between the following scales: nausea and vomiting (QLQ-C30) and arm symptoms (0.07); constipation (QLQ-C30) and future perspective (0.03); and diarrhoea (QLQ-C30) and breast symptoms (0.08) (Table 3).

**Table 3:** Evaluation of convergent and discriminant validity. *Correlations between QLQ-BR42 Scales and QLQ-C30. Spearman Correlation*

	Body Image	Sexual Function	Sexual Enjoyment	Future Perspective	Breast Satisfaction	Arm Symptoms	Breast Symptoms	Systemic Chemo Side Effects 8 items	Vaginal Symptoms	Endocrine Symptoms	Hand/feet symptoms/neuropathy	Weight Gain	Skeletal
QLQ-C30													
Physical functioning	0.232	0.243	0.212	0.282	0.232	-0.288	-0.085	-0.463	-0.346	-0.188	-0.290	-0.269	-0.504a
Role functioning	0.314	0.138	0.157	0.365	0.290	-0.270	-0.156	-0.269	-0.382	-0.314	-0.256	-0.229	-0.389
Social Functioning	0.132	0.022	0.113	0.366	0.166	-0.121	-0.132	-0.193	-0.247	-0.138	-0.138	-0.113	-0.212
Emotional Functioning	0.187	0.044	0.171	0.145	0.153	-0.231	-0.159	-0.352	-0.380	-0.140	-0.280	-0.138	-0.363
Cognitive functioning	0.416	0.102	0.131	0.236	0.258	-0.187	-0.202	-0.292	-0.379	-0.304	-0.232	-0.301	-0.291
Global Quality of Life Scale	0.260	0.161	0.115	0.317	0.299	-0.238	-0.155	-0.415	-0.360	-0.250	-0.252	-0.307	-0.419
Fatigue Scale	-0.344	-0.235	-0.294	-0.419	-0.318	0.328	0.154	0.408	0.530	0.324	0.324	0.323	0.511
Nausea / Vomiting Scale	-0.206	-0.146	-0.076	-0.122	-0.093	0.070b	0.164	0.148	0.365	0.227	0.117	0.082	0.095
Pain Scale	-0.213	-0.111	-0.220	-0.283	-0.161	0.272	0.197	0.384	0.324	0.240	0.399	0.235	0.567a
Dyspnoea Scale	-0.279	-0.033	-0.133	-0.278	-0.219	0.189	0.221	0.230	0.121	0.365	0.102	0.110	0.100
Sleep Disturbances	-0.136	-0.032	-0.053	-0.327	-0.300	0.157	0.202	0.198	0.268	0.258	0.138	0.106	0.274
Appetite Loss	-0.189	-0.035	-0.212	-0.091	-0.172	0.089	0.092	0.232	0.190	0.156	0.190	0.225	0.232
Constipation Scale	-0.129	-0.084	-0.191	-0.033b	-0.154	0.121	0.110	0.191	0.262	0.177	0.222	0.205	0.290
Diarrhoea Scale	-0.162	-0.029	-0.057	-0.190	-0.164	0.176	0.077b	0.162	0.399	0.085	0.148	0.147	0.211
Financial Impact	-0.179	-0.042	-0.121	-0.139	-0.263	0.153	0.136	0.183	0.305	0.265	0.096	0.340	0.223

a: higher correlations were hypothesised

b: lower correlations were hypothesised

### Known-groups validity

Results of the known-groups comparisons are shown in Table 4. QOL scores were higher in five QLQ-BR42 areas among patients from the ET cohort (small effect sizes in two areas, moderate in one, and large in two; Cohen’s *d*) [37]. No significant differences were observed within the ET cohort according to breast surgery modality. Patients who underwent mastectomy showed more arm symptoms (*p* = 0.092, small effect size, Cohen’s *d* = 0.32).

**Table 4:** Evaluation of construct validity based on known groups. *Known groups. Mean (SD)*

	*N*	Body Image	Sexual Function	Sexual Enjoyment	Future Perspective	Breast Satisfaction	Arm Symptoms	Breast Symptoms	Systemic Chemo Side Effects	Vaginal Symptoms	Endocrine Symptoms	Hand/foot symptoms/neuropathy	Weight Gain	Skeletal
Cohorts														
ET		92.4 (15.8)	16.1 (19.8)	33.3 (22.4)	71.3 (30.8)	79.8 (18.6)	14.2 (19.9)	9.4 (14.1)	14.9 (12.7)	5.1 (9.8)	13.5 (17.9)	21.8 (19.5)	16.0 (20.5)	36.6 (27.0)
Metastases		88.0 (23.3)	18.1 (19.1)	44.9 (25.8)	67.3 (33.8)	59.5 (30.2)	21.5 (25.9)	11.1 (17.2)	20.2 (16.0)	0.0 (0.0)	18.5 (16.9)	25.5 (27.4)	45.2 (32.6)	44.6 (31.0)
*P*-value		0.101	0.554	0.069	0.344	0.000	0.020	0.398	0.027	0.018	0.078	0.348	0.000	0.091
ES		0.22	0.10	0.48	0.12	0.81	0.32	0.11	0.32	0.73	0.29	0.16	1.07	0.27
Only ET cohort														
Surgery														
Conservative		93.9 (14.2)	15.2 (19.8)	36.2 (18.7)	73.3 (28.5)	81.2 (17.7)	12.6 (18.7)	8.0 (13.1)	14.6 (13.5)	4.9 (9.0)	12.5 (16.9)	20.4 (19.2)	15.5 (19.0)	34.4 (26.0)
Radical		89.6 (17.4)	16.7 (20.1)	29.2 (29.5)	68.2 (35.9)	76.7 (20.4)	19.2 (22.5)	12.7 (16.4)	15.6 (10.9)	5.6 (11.9)	15.8 (20.3)	25.0 (20.3)	17.3 (23.8)	41.7 (29.0)
*P*-value		0.147	0.737	0.392	0.398	0.337	0.092	0.097	0.734	0.887	0.484	0.342	0.733	0.287
ES		0.22	0.08	0.37	0.16	0.24	0.32	0.32	0.08	0.07	0.18	0.23	0.08	0.26

*ET* cohort in which endocrine treatment was studied

*ES* effect size, Cohen’s d

### Responsiveness to change

Significant differences between the two assessments were observed in three QLQ-BR42 scales among patients whose global QOL improved, with small effect sizes (Cohen’s *d* = 0.44–0.49). In patients whose global QOL worsened, a significant difference was observed in the arm symptoms scale (fewer symptoms, small effect size) [37]. In five QLQ-BR42 scales, no significant differences were observed between the two assessments, although the trend was towards worsening at the second assessment: effect sizes were large (> 1.0) in two scales, moderate in one (0.76), and small in the remaining two (0.24 and 0.38) (Supplementary Tables 7 and 8).

## Discussion

We have presented the results of a validation study of the EORTC QLQ-BR42 for Spain. The results from this first national psychometric study of this questionnaire complement those from the international validation study. The sample characteristics – including more than 10 patients per item [30] and variability in the demographic (e.g., age, cohabitation) and clinical variables (e.g., disease stage, present and past treatment modalities) – were adequate for this study.

Levels of compliance were good, with no or few missing data in most items, which indicates that the instrument was well accepted by patients. Exceptions were observed in four items conditional on having experienced hair loss or being sexually active, as well as in two items from the sexual functioning scale. Low levels of compliance (and low scores) for the sexual functioning items have also been reported in other studies conducted by our group with BC patients [38, 39]. The moderately high mean age (65.1) may have contributed to these low compliance rates and mean scores: the patients who failed to respond to either of the two sexual functioning items in our study were older than those who replied to both items; indeed, Ng et al. [40] also found that older participants were more likely to omit these sexuality items.

The structure of the questionnaire was generally confirmed by confirmatory factor analyses, with satisfactory goodness of fit measures. Seven scales whose structures mostly aligned with the proposed QLQ-BR42 structure were found in the model. Some items were excluded from the analyses due to missing responses. These include sexual functioning, which, like sexual enjoyment, is a key QOL area [41], and vaginal symptoms, as reported in the international study [14]. Vaginal symptoms may be more strongly associated with endocrine treatment than with chemotherapy [42].

Three items that also address key QOL areas were not included because they were individual items: sexual enjoyment, future perspective (which is a relevant QOL area for breast cancer, especially in advanced disease stages [43]), and weight gain, which may be mainly related to chemotherapy [44].

Four items, two of which were lower and two were higher, had different loadings from those in the EORTC international validation study. Only two items did not fit the proposed model: item 40 (feeling less feminine) from the body image scale (most content on this body image scale was retained in the model); and item 71 (satisfaction with the cosmetic result of surgery) from the breast satisfaction scale. This latter item addresses an important QOL issue [45]. The structure of these two scales should be assessed in future studies.

Limitations in the systemic chemotherapy side effects domain were observed in three analyses (factor analyses, Cronbach’s alpha, and convergent and discriminant validity). These findings may be related to the distribution of scores, as most items were answered in the lowest categories. The low scores likely reflect the fact that only 27% of the sample received CT and, of these, 44.4% (the ET subsample) received CT roughly five years before the first QOL assessment, when toxicity may have been lower. This scale met the reliability criteria and factor loadings were higher in the international validation study which, unlike ours, covered the full range of scores (0-100) [14].

The distribution of the scores across the different scales was satisfactory, with most scales exhibiting the full range of scores. Floor and ceiling effects were moderate, reflecting generally good QOL, which is consistent with most patients being in early disease stages. Reliability analyses were satisfactory, with all but one scale (the systemic chemotherapy side effects scale) meeting the Cronbach’s alpha criteria.

Test re-test reliability was good among patients whose overall health status remained stable.

Convergent and discriminant validity results were generally positive. Item scale correlations were satisfactory for most items. Correlations with the QLQ-C30 were also satisfactory, with higher correlations observed in areas whose contents were more related and lower correlations in areas whose contents were less related. Overall, correlations were not high, which indicates that the QLQ-BR42 assesses different concepts from those measured by the QLQ-C30.

Known-groups validity was satisfactory, as QLQ scores were higher in the ET cohort than in the metastatic group. This likely reflects the fact that ET patients were in earlier disease stages and in long-term follow-up after completing RT or CT, whereas the metastatic group may have been receiving active treatment. Some similarities (in systemic chemotherapy side effects) were found in significant areas between the EORTC international validation study [14] and ours. However, some differences were also found, perhaps due to the different disease stages of the groups compared. Consistent with other studies (e.g., Tsai et al. [46] on *body image*), differences between breast surgery modalities were observed in a limited number of areas (e.g., *arm symptoms*), with the differences favouring patients who underwent breast-conserving surgery.

Responsiveness to changes was assessed in two small patient samples. Despite the limited sample sizes, trends towards improvements or deterioration in QOL scores were observed among patients whose global QOL scores increased or decreased. These trends suggest that the questionnaire may be sensitive to changes in patient health status. However, these results should be considered preliminary and require more detailed study. More significant differences were found in the international validation study [14], but the patients in that study had received oncological treatment between the two assessments.

The strengths of this study are the large patient sample and the representation of different disease stages, treatment stages and treatment modalities. However, the study also has certain limitations. For example, this study was performed in just one centre. Also, the age distribution and high mean age may have influenced both the large number of missing responses on the sexuality items and the sexuality scales analyses. Moreover, the mixed timing in relation to treatment exposure in the three samples may have affected symptoms scores, while item exclusions in CFA may have reduced comparability with the full structure of the QLQ-BR42 questionnaire. Responsiveness to change could have been evaluated in a larger sample of patients who received treatment between the two assessments in order to determine whether the trends found in the present study were confirmed and whether more significant changes were found in other areas. Similarly, test-retest reliability could have been assessed using two measurements that were closer in time (approximately two weeks) to ensure that the patients’ conditions remained stable [32].

## Conclusions

The EORTC QLQ-BR42 questionnaire has demonstrated satisfactory psychometric properties when applied to a sample of Spanish BC patients. Future studies could focus on younger patients from other regions of the country in order to assess compliance with the sexuality items. They could also focus on patients receiving active chemotherapy to evaluate the psychometric functioning of the systemic chemotherapy side effects scale, the overall scale structure, and the responsiveness to change.

## Supplementary Information

Supplementary Material 1.

Supplementary Material 2.

Supplementary Material 3.

Supplementary Material 4.

Supplementary Material 5.

## Data Availability

The datasets used and/or analysed during this study are available from the corresponding author on reasonable request.

## Abbreviations

QOL: Quality of life
BC: Breast cancer
BCSs: Breast cancer survivors
EORTC: European Organisation for Research and Treatment of Cancer
QLG: Quality of Life Group
TT: Targeted Therapy
ET: Endocrine Treatment
RT: Radiotherapy
CT: Chemotherapy
CFI: Comparative Fit Index
TLI: Tucker-Lewis Index) RMSEA (Root Mean Square Error of Approximation
