# Supplementary material for: The EORTC updated breast cancer quality of life questionnaire EORTC QLQ-BR42: A psychometric study with Spanish patients
Source: BMC Cancer. 2026 Mar 14;26:522. doi: 10.1186/s12885-026-15831-8 (PMC13107805; doi:10.1186/s12885-026-15831-8)
Supplement: Supplementary file 1 — Supplementary Material 1. [file 12885_2026_15831_MOESM1_ESM.docx]

**Supplementary Table 1. General questionnaire and breast cancer module**

| **General EORTC questionnaire QLQ-C30** |  |
| --- | --- |
| Functional scales (1) | Physical, role, cognitive, emotional, social, global quality of life. |
| Symptom scales and/or items (2) | Fatigue, nausea and vomiting, pain. Individual items on dyspnoea, sleep disturbance, appetite loss, constipation, diarrhoea, financial impact. |
| **EORTC module QLQ-BR42** |  |
| Functioning scales and/or items (1) | Body image, sexual functioning, breast satisfaction. Individual items on sexual enjoyment, future perspective. |

| Symptoms scales and/or items (2) | Systemic chemotherapy side effects, arm symptoms, breast symptoms, endocrine symptoms, vaginal symptoms, hand/feet symptoms/neuropathy, weight gain, skeletal symptoms. |
| --- | --- |

1. Scores range from 0 to 100, with higher scores representing higher functional levels.

2. Scores range from 0 to 100, with higher scores representing greater degrees of symptoms.
