# Supplementary material for: The EORTC updated breast cancer quality of life questionnaire EORTC QLQ-BR42: A psychometric study with Spanish patients
Source: BMC Cancer. 2026 Mar 14;26:522. doi: 10.1186/s12885-026-15831-8 (PMC13107805; doi:10.1186/s12885-026-15831-8)
Supplement: Supplementary file 2 — Supplementary Material 2. [file 12885_2026_15831_MOESM2_ESM.docx]

Supplementary Table 2

Scale structure of the QLQ-BR42 and frequencies of item responses

| Scale | Item | | | Reason for exclusion | Number of available responses  N (%) | Number of missing responses |
| --- | --- | --- | --- | --- | --- | --- |
| Body Image |  | | |  |  |  |
|  | BR39 | | |  | 516 (100%) | 0 |
|  | *BR40 | | | Did not fit the model | 516 (100%) | 0 |
|  | BR41 | | |  | 516 (100%) | 0 |
|  | BR42 | | |  | 516 (100%) | 0 |
| Sexual Function |  | | |  |  |  |
|  | *BR44 | | | Component of the sexual functioning scale  High percentage of missing responses | 310 (60.1%) | 206 |
|  | *BR45 | | | No patient responded with the highest score  High percentage of missing responses | 290 (56.2%) | 226 |
| Breast Satisfaction |  | | |  |  |  |
|  | *BR71 | | | Did not fit the model | 515 (99.8%) | 1 |
|  | *BR72 | | | Component of the breast satisfaction scale | 515(99.8%) | 1 |
| Arm Symptoms |  | | |  |  |  |
|  | BR47 | | |  | 511 (99.1%) | 5 |
|  | BR48 | | |  | 516 (100%) | 0 |
|  | | BR49 |  | | 516 (100%) | 0 |
| Breast Symptoms | |  |  | |  |  |
|  | | BR50 | |  | 514 (99.6%) | 2 |
|  | | BR51 | |  | 516 (100%) | 0 |
|  | | BR52 | |  | 516 (100%) | 0 |
|  | | BR53 | |  | 516 (100%) | 0 |
| Systemic Chemo SE | |  |  | |  |  |
|  | | BR31 | |  | 516 (100%) | 0 |
|  | | BR32 | |  | 516 (100%) | 0 |
|  | | BR33 | |  | 516 (100%) | 0 |
|  | | BR34 | |  | 513 (99.4%) | 3 |
| Loss Hair | | *BR35 | | High percentage of missing responses | 143 (27.7%) | 373 |
|  | | BR36 | |  | 511 (99.1%) | 5 |
|  | | BR57 | |  | 516 (100%) | 0 |
|  | | BR58 | |  | 516 (100%) | 0 |
| Vaginal Symptoms | |  | |  |  |  |
|  | | *BR68 | | Component of the vaginal symptoms scale | 431 (83.5%) | 85 |
|  | | *BR69 | | High percentage of missing responses | 209 (40.5%) | 307 |
|  | | *BR70 | | High percentage of missing responses | 209 (40.5%) | 307 |
| Endocrine Symptoms | |  |  | |  |  |
|  | | BR37 |  | | 514 (99.6%) | 2 |
|  | | BR38 |  | | 516 (100%) | 0 |
|  | | BR54 |  | | 516 (100%) | 0 |
|  | | BR55 |  | |  | 0 |
|  | | BR56 |  | | 513 (99.4%) | 3 |
| Hand/foot symptoms/neuropathy | |  |  | |  |  |
|  | | BR59 |  | | 516 (100%) | 0 |
|  | | BR60 |  | | 516 (100%) | 0 |
|  | | BR61 |  | | 516 (100%) | 0 |
|  | | BR62 |  | | 516 (100%) | 0 |
| Skeletal Symptoms | |  |  | |  |  |
|  | | BR63 |  | | 516 (100%) | 0 |
|  | | BR64 |  | | 516 (100%) | 0 |
|  | | BR65 |  | | 516 (100%) | 0 |
|  | | BR66 |  | | 516 (100%) | 0 |
| **Individual items** | |  |  | |  |  |
| Future Perspective | | BR43 | Individual | | 513 (99.4%) | 3 |
| Sexual Enjoyment | | BR46 | Individual | | 172 (33.3%) | 344 |
| Weight Gain | | BR67 | Individual | | 516 (100%) | 0 |

Scale structure of the QLQ-BR42 based on Confirmatory Factor Analysis, reasons for item exclusion, and numbers of available and missing responses.

Scale: QLQ-BR42 scale structure.

Item: items in each scale, both before and after applying the Confirmatory Factor Analyses model.

*Items excluded from the final Confirmatory Factor Analyses model.

Reasons for exclusion: reason for exclusion from the final Confirmatory Factor Analyses model.
