# Supplementary material for: The EORTC updated breast cancer quality of life questionnaire EORTC QLQ-BR42: A psychometric study with Spanish patients
Source: BMC Cancer. 2026 Mar 14;26:522. doi: 10.1186/s12885-026-15831-8 (PMC13107805; doi:10.1186/s12885-026-15831-8)
Supplement: Supplementary file 3 — Supplementary Material 3. [file 12885_2026_15831_MOESM3_ESM.docx]

Supplementary Table 3

Comparison of patients with no missing response(s) and some missing response(s) in the Sexual Functioning scale

|  | **Patients with no missing responses in** **Sexual Functioning**  **(n = 290)** | **Patients** **with missing response(s) in Sexual Functioning**  **(n = 226)** | **p-value** |
| --- | --- | --- | --- |
| **SOCIODEMOGRAPHIC** |  |  |  |
| **Age, mean (SD)** | 64.0 (9.8) | 66.6 (10.1) | 0.004 |
| **Marital status, n (%)** |  |  |  |
| Single | 21 (7.3%) | 32 (14.4%) | < 0.001 |
| Married | 246 (85.4%) | 135 (60.8%) |  |
| Separated | 8 (2.8%) | 22 (9.9%) |  |
| Widowed | 13 (4.5%) | 33 (14.9%) |  |
| **Cohabitation, n (%)** |  |  |  |
| Alone | 23 (8.1%) | 42 (19.7%) | < 0.001 |
| With a partner | 179 (63.3%) | 105 (49.3%) |  |
| With children over 18 | 72 (25.4%) | 48 (22.5%) |  |
| With other people | 9 (3.2%) | 18 (8.4%) |  |
| **CLINICAL** |  |  |  |
| **Karnofsky, mean (SD)**  Range | 81.9 (8.9)  50-100 | 77.7 (8.4)  50-100 | < 0.001 |
| **Karnofsky 3 categories, n (%)** |  |  |  |
| 90 – 100 | 111 (39.6%) | 33 (15.2%) | < 0.001 |
| 80 | 110 (39.3%) | 113 (52.1%) |  |
| 50 – 70 | 59 (21.1%) | 71 (32.7%) |  |
| **Breast surgery n (%)** |  |  |  |
| Conservative | 153 (54.4%) | 105 (47.5%) | 0.123 |
| Radical | 128 (45.6%) | 116 (52.5%) |  |
| **Axillary surgery, n (%)** |  |  |  |
| Axillary node dissection | 64 (23.1%) | 43 (20.3%) | 0.460 |
| Sentinel node biopsy | 204 (73.6%) | 165 (77.8%) |  |
| No | 9 (3.2%) | 4 (1.9%) |  |
| **TREATMENT (past or present)** |  |  |  |
| **Hormonotherapy** |  |  |  |
| No | 10 (3.9%) | 14 (7.5%) | 0.246 |
| Yes | 119 (46.3%) | 86 (46.0%) |  |
| **Chemotherapy** |  |  |  |
| No | 221 (76.1%) | 182 (84.3%) | 0.083 |
| Yes | 62 (21.9%) | 34 (15.7%) |  |
| **Radiotherapy** |  |  |  |
| No | 42 (14.9%) | 52 (24.1%) | 0.010 |
| Yes | 239 (85.1%) | 164 (75.9%) |  |
| **COMORBIDITY** |  |  |  |
| **Any** | 69 (24.9%) | 45 (20.5%) | 0.252 |

Comparison of the demographic and clinical characteristics of patients who responded to the two sexual functioning items and those who had at least one missing response on this scale.
