# Supplementary material for: The EORTC updated breast cancer quality of life questionnaire EORTC QLQ-BR42: A psychometric study with Spanish patients
Source: BMC Cancer. 2026 Mar 14;26:522. doi: 10.1186/s12885-026-15831-8 (PMC13107805; doi:10.1186/s12885-026-15831-8)
Supplement: Supplementary file 4 — Supplementary Material 4. [file 12885_2026_15831_MOESM4_ESM.docx]

#### **Supplementary Table 4. Standardised Regression Weights (SRW) of the CFA model**

| Scales | SRW | Variance |
| --- | --- | --- |
| Body image |  |  |
| BR41 | 0.835 | 0.302 |
| BR42 | 0.875 | 0.235 |
| BR39 | 0.989 | 0.022 |
| Arm symptoms |  |  |
| BR47 | 0.593 | 0.648 |
| BR48 | 0.977 | 0.045 |
| BR49 | 0.858 | 0.264 |
| Breast symptoms |  |  |
| BR50 | 0.848 | 0.281 |
| BR51 | 0.893 | 0.203 |
| BR52 | 0.859 | 0.262 |
| BR53 | 0.651 | 0.577 |
| Systemic chemotherapy side effects |  |  |
| BR31 | 0.292 | 0.915 |
| BR32 | 0.728 | 0.469 |
| BR33 | 0.405 | 0.836 |
| BR34 | 0.304 | 0.907 |
| BR36 | 0.732 | 0.464 |
| BR57 | 0.688 | 0.527 |
| BR58 | 0.593 | 0.648 |
| Endocrine symptoms |  |  |
| BR54 | 0.661 | 0.563 |
| BR55 | 0.713 | 0.491 |
| BR56 | 0.729 | 0.469 |
| BR37 | 0.681 | 0.536 |
| BR38 | 0.608 | 0.630 |
| Hand/foot symptoms/neuropathy |  |  |
| BR59 | 0.656 | 0.570 |
| BR60 | 0.638 | 0.593 |
| BR61 | 0.884 | 0.218 |
| BR62 | 0.936 | 0.124 |
| Skeletal symptoms |  |  |
| BR63 | 0.954 | 0.090 |
| BR64 | 0.855 | 0.270 |
| BR65 | 0.915 | 0.162 |
| BR66 | 0.813 | 0.340 |

BR31 to BR66: the QLQ-BR42 items.
