# Supplementary material for: The EORTC updated breast cancer quality of life questionnaire EORTC QLQ-BR42: A psychometric study with Spanish patients
Source: BMC Cancer. 2026 Mar 14;26:522. doi: 10.1186/s12885-026-15831-8 (PMC13107805; doi:10.1186/s12885-026-15831-8)
Supplement: Supplementary file 5 — Supplementary Material 5. [file 12885_2026_15831_MOESM5_ESM.docx]

#### **Supplementary Table 5.** Test-retest reliability

#### Only patients whose status remained stable in the global QOL item between the second and third evaluations. ET cohort

| **ET cohort** | Second evaluation  Mean (SD) | Third evaluation  Mean (SD) | Change  Mean (SD) * | p-value | ICC | 95% IC |
| --- | --- | --- | --- | --- | --- | --- |
| Body Image ^a^ | 93.0 (14.6) | 93.0 (15.6) | 0.0 (7.7) | 1.000 | 0.868 | 0.813 ; 0.908 |
| Sexual Function ^a^ | 18.1 (19.4) | 20.7 (21.5) | 2.6 (12.2) | 0.078 | 0.821 | 0.727 ; 0.885 |
| Sexual Enjoyment ^a^ | 51.0 (20.7) | 46.9 (20.5) | -4.2 (20.3) | 0.255 | 0.515 | 0.207 ; 0.729 |
| Future Perspective ^a^ | 71.9 (23.4) | 62.7 (30.3) | -9.2 (21.7) | < 0.001 | 0.680 | 0.560 ; 0.772 |
| Breast Satisfaction ^a^ | 79.1 (24.2) | 79.3 (23.7) | 0.2 (4.3) | 0.658 | 0.984 | 0.974 ; 0.990 |
| Arm Symptoms ^b^ | 11.5 (18.2) | 9.3 (15.4) | -2.2 (9.7) | 0.017 | 0.836 | 0.769 ; 0.885 |
| Breast Symptoms ^b^ | 7.7 (11.1) | 7.9 (11.7) | 0.2 (6.6) | 0.712 | 0.831 | 0.760 ; 0.882 |
| Systemic Chemotherapy Side Effects ^b^ | 11.8 (11.5) | 12.2 (12.5) | 0.4 (4.3) | 0.430 | 0.935 | 0.899 ; 0.958 |
| Vaginal Symptoms ^b^ | 2.9 (6.2) | 2.9 (6.2) | 0.0 (0.0) | --- | 1.000 | --- |
| Endocrine Symptoms ^b^ | 10.0 (12.1) | 11.9 (15.5) | 2.0 (5.3) | 0.002 | 0.927 | 0.888 ; 0.953 |
| Hand/foot symptoms/neuropathy ^b^ | 17.1 (16.8) | 24.3 (22.2) | 7.2 (14.8) | < 0.001 | 0.717 | 0.590 ; 0.809 |
| Weight Gain ^b^ | 13.4 (20.4) | 14.3 (22.6) | 0.9 (7.6) | 0.320 | 0.938 | 0.904 ; 0.960 |
| Skeletal ^b^ | 27.5 (20.6) | 29.4 (22.6) | 1.9 (7.8) | 0.033 | 0.935 | 0.901 ; 0.958 |

* Negative values indicate worsening.

a. Scores range from 0 to 100, with higher scores representing higher functional levels.

b. Scores range from 0 to 100, with higher scores representing greater degrees of symptoms.

#### **Supplementary Table 6. Evaluation of convergent and discriminant validity**

*Item-correlation scale (Spearman Correlation)* ***excluding the item when calculating the scale*** *and Cronbach's alpha after removing the item.*

|  | Body Image | Sexual  Function | Sexual  Enjoyment | Future  Perspective | Breast  Satisfaction | Arm  Symptoms | Breast  Symptoms | Syst. Chemo  Side Effects | Vaginal  Symptoms | Endocrine  Symptoms | Skin  Toxicity | Weight  Gain | Skeletal | Cronbach's alpha * |
| --- | --- | --- | --- | --- | --- | --- | --- | --- | --- | --- | --- | --- | --- | --- |
| Body Image |  |  |  |  |  |  |  |  |  |  |  |  |  |  |
| Item 39 | **0.812** | -0.137 | -0.326 | -0.357 | -0.306 | 0.103 | 0.277 | 0.281 | 0.225 | 0.371 | 0.209 | 0.273 | 0.190 | 0.83 |
| Item 40 | **0.688** | -0.048 | -0.285 | -0.385 | -0.332 | 0.208 | 0.379 | 0.317 | 0.276 | 0.384 | 0.232 | 0.300 | 0.207 | 0.83 |
| Item 41 | **0.688** | -0.154 | -0.167 | -0.268 | -0.296 | 0.142 | 0.149 | 0.152 | 0.339 | 0.262 | 0.120 | 0.157 | 0.139 | 0.87 |
| Item 42 | **0.817** | 0.008 | -0.235 | -0.362 | -0.355 | 0.152 | 0.256 | 0.299 | 0.235 | 0.309 | 0.250 | 0.218 | 0.241 | 0.87 |
| Sexual Function |  |  |  |  |  |  |  |  |  |  |  |  |  |  |
| Item 44 | 0.070 | **0.700** | 0.376 | -0.156 | 0.153 | 0.005 | 0.165 | -0.075 | -0.271 | 0.109 | -0.048 | -0.034 | -0.088 | --- |
| Item 45 | 0.193 | **0.700** | 0.612 | -0.071 | 0.292 | 0.063 | -0.017 | -0.295 | -0.125 | -0.174 | -0.264 | -0.266 | -0.328 | --- |
| Breast Satisfaction |  |  |  |  |  |  |  |  |  |  |  |  |  |  |
| Item 71 | 0.392 | 0.201 | 0.148 | 0.238 | **0.408** | -0.232 | -0.188 | -0.143 | -0.159 | -0.240 | -0.240 | -0.324 | -0.277 | --- |
| Item 72 | 0.387 | 0.199 | 0.094 | 0.159 | **0.408** | -0.184 | -0.223 | -0.118 | -0.226 | -0.235 | -0.267 | -0.312 | -0.281 | --- |
| Arm Symptoms |  |  |  |  |  |  |  |  |  |  |  |  |  |  |
| Item 47 | -0.115 | 0.065 | 0.011 | -0.268 | -0.128 | **0.653** | 0.298 | 0.215 | 0.328 | 0.264 | 0.264 | 0.097 | 0.275 | 0.59 |
| Item 48 | -0.201 | -0.001 | -0.036 | -0.241 | -0.311 | **0.476** | 0.319 | 0.203 | 0.197 | 0.200 | 0.292 | 0.234 | 0.311 | 0.78 |
| Item 49 | -0.218 | -0.097 | -0.075 | -0.185 | -0.208 | **0.637** | 0.289 | 0.296 | 0.029 | 0.333 | 0.247 | 0.257 | 0.272 | 0.62 |
| Breast Symptoms |  |  |  |  |  |  |  |  |  |  |  |  |  |  |
| Item 50 | -0.275 | 0.047 | -0.070 | -0.146 | -0.238 | 0.257 | **0.524** | 0.081 | 0.319 | 0.137 | 0.237 | 0.129 | 0.246 | 0.63 |
| Item 51 | -0.251 | -0.042 | 0.131 | -0.113 | -0.144 | 0.238 | **0.452** | 0.222 | -0.112 | 0.177 | 0.137 | 0.240 | 0.088 | 0.66 |
| Item 52 | -0.201 | 0.225 | -0.150 | -0.247 | -0.129 | 0.206 | **0.562** | 0.127 | 0.216 | 0.275 | 0.247 | 0.150 | 0.217 | 0.61 |
| Item 53 | -0.232 | -0.035 | -0.178 | -0.218 | -0.096 | 0.266 | **0.424** | 0.093 | 0.423 | 0.211 | 0.228 | 0.118 | 0.098 | 0.67 |
| Syst. Chemo Side Effects |  |  |  |  |  |  |  |  |  |  |  |  |  |  |
| Item 31 | -0.087 | -0.088 | -0.072 | -0.246 | 0.035 | 0.241 | 0.060 | **0.364** | -0.087 | 0.168 | 0.168 | 0.114 | 0.317 | 0.51 |
| Item 32 | -0.214 | 0.087 | -0.278 | -0.195 | -0.197 | 0.200 | 0.163 | 0.057 | **0.434** | 0.171 | 0.229 | 0.191 | 0.268 | 0.53 |
| Item 33 | -0.135 | -0.082 | 0.030 | -0.103 | -0.036 | 0.093 | 0.039 | 0.264 | 0.069 | 0.210 | 0.218 | **0.268** | 0.235 | 0.51 |
| Item 34 | -0.112 | -0.074 | -0.171 | -0.057 | -0.073 | 0.085 | 0.116 | 0.108 | 0.075 | 0.146 | 0.075 | 0.086 | **0.133** | 0.47 |
| Item 35 | -0.051 | **0.198** | 0.087 | -0.192 | 0.072 | -0.023 | 0.086 | 0.182 | -0.063 | 0.072 | 0.055 | -0.044 | 0.008 | 0.56 |
| Item 36 | -0.389 | -0.148 | -0.144 | -0.323 | -0.261 | 0.228 | 0.214 | 0.126 | **0.357** | 0.298 | 0.241 | 0.306 | 0.362 | 0.50 |
| Item 57 | -0.152 | -0.011 | -0.215 | -0.194 | -0.207 | 0.236 | 0.209 | 0.257 | 0.140 | 0.304 | 0.198 | 0.372 | **0.331** | 0.49 |
| Item 58 | -0.206 | -0.078 | -0.221 | -0.024 | -0.054 | 0.133 | 0.063 | 0.102 | . | **0.333** | 0.247 | 0.214 | 0.222 | 0.52 |
| Vaginal Symptoms |  |  |  |  |  |  |  |  |  |  |  |  |  |  |
| Item 68 | -0.242 | -0.040 | -0.338 | -0.148 | -0.087 | 0.046 | 0.103 | 0.284 | **0.777** | 0.280 | 0.268 | 0.281 | 0.281 | 0.91 |
| Item 69 | -0.422 | -0.266 | -0.335 | -0.416 | -0.190 | 0.415 | 0.417 | 0.017 | **0.797** | 0.226 | 0.484 | 0.074 | 0.509 | 0.89 |
| Item 70 | -0.389 | -0.171 | -0.378 | -0.359 | -0.161 | 0.251 | 0.295 | 0.218 | **0.897** | 0.191 | 0.438 | 0.146 | 0.434 | 0.81 |
| Endocrine Symptoms |  |  |  |  |  |  |  |  |  |  |  |  |  |  |
| Item 37 | -0.351 | 0.094 | -0.207 | -0.303 | -0.109 | 0.187 | 0.270 | 0.278 | -0.033 | **0.496** | 0.176 | 0.243 | 0.215 | 0.65 |
| Item 38 | -0.236 | -0.009 | -0.198 | -0.290 | -0.115 | 0.153 | 0.159 | 0.308 | 0.115 | **0.472** | 0.229 | 0.091 | 0.229 | 0.65 |
| Item 54 | -0.173 | 0.116 | 0.009 | -0.226 | -0.156 | 0.175 | 0.230 | 0.187 | 0.176 | **0.591** | 0.319 | 0.366 | 0.285 | 0.60 |
| Item 55 | -0.301 | -0.038 | -0.017 | -0.170 | -0.234 | 0.134 | 0.220 | 0.224 | 0.168 | **0.419** | 0.259 | 0.401 | 0.267 | 0.66 |
| Item 56 | -0.216 | -0.057 | -0.150 | -0.257 | -0.240 | 0.126 | 0.047 | 0.345 | 0.035 | 0.352 | 0.297 | 0**.360** | 0.358 | 0.69 |
| Hand/feet symptoms/neuropathy |  |  |  |  |  |  |  |  |  |  |  |  |  |  |
| Item 59 | -0.210 | 0.117 | -0.130 | -0.301 | -0.105 | 0.224 | 0.179 | 0.235 | 0.405 | 0.208 | 0.452 | 0.136 | **0.523** | 0.71 |
| Item 60 | -0.120 | -0.162 | 0.056 | -0.086 | -0.267 | 0.221 | 0.143 | 0.085 | 0.283 | 0.273 | **0.379** | 0.278 | 0.305 | 0.74 |
| Item 61 | -0.226 | -0.113 | -0.323 | -0.180 | -0.246 | 0.248 | 0.228 | 0.236 | 0.379 | 0.274 | **0.639** | 0.242 | 0.510 | 0.60 |
| Item 62 | -0.305 | -0.179 | -0.296 | -0.149 | -0.269 | 0.294 | 0.246 | 0.294 | 0.444 | 0.357 | **0.631** | 0.289 | 0.607 | 0.59 |
| Skeletal |  |  |  |  |  |  |  |  |  |  |  |  |  |  |
| Item 63 | -0.299 | -0.159 | -0.339 | -0.352 | -0.242 | 0.305 | 0.188 | 0.424 | 0.318 | 0.338 | 0.639 | 0.329 | **0.810** | 0.84 |
| Item 64 | -0.240 | -0.292 | -0.217 | -0.264 | -0.260 | 0.201 | 0.192 | 0.334 | 0.375 | 0.267 | 0.500 | 0.352 | **0.707** | 0.88 |
| Item 65 | -0.304 | -0.218 | -0.245 | -0.239 | -0.306 | 0.252 | 0.148 | 0.398 | 0.354 | 0.316 | 0.624 | 0.380 | **0.819** | 0.84 |
| Item 66 | -0.163 | -0.064 | -0.181 | -0.190 | -0.250 | 0.331 | 0.203 | 0.362 | 0.387 | 0.345 | 0.573 | 0.421 | **0.717** | 0.88 |

**Cells in grey:** correlation item own scale (corrected for overlap).

**Numbers in bold:** Highest correlation of each item with its own scale and with other scales.

**Supplementary Table 7**. Sensitivity to change according to the hypothesis of improvement in the global QOL item

*Only patients whose global QOL item improved between the first and second evaluations. ET cohort (n = 33)*

| **ET cohort** | First evaluation  Mean (SD) | Second evaluation  Mean (SD) | Change*  Mean (SD) | *p-*value | ES | 95% IC |
| --- | --- | --- | --- | --- | --- | --- |
| Body Image ^a^ | 91.9 (10.7) | 93.4 (10.6) | 1.5 (4.9) | 0.083 | 0.14 | -0.20 ; 0.48 |
| Sexual Function ^a^ | 23.3 (19.0) | 17.5 (19.8) | -5.8 (15.6) | 0.110 | -0.31 | -0.66 ; 0.05 |
| Sexual Enjoyment ^a^ | 33.3 (17.8) | 37.5 (11.8) | 4.2 (21.4) | 0.598 | 0.24 | -0.11 ; 0.59 |
| Future Perspective ^a^ | 58.6 (27.7) | 70.7 (26.0) | 12.1 (23.3) | 0.005 | 0.44 | 0.06 ; 0.81 |
| Breast Satisfaction ^a^ | 69.4 (17.5) | 75.0 (18.4) | 5.6 (16.1) | 0.103 | 0.32 | -0.04 ; 0.68 |
| Arm Symptoms ^b^ | 23.9 (25.2) | 22.9 (25.1) | 1.0 (14.5) | 0.692 | 0.04 | -0.38 ; 0.30 |
| Breast Symptoms ^b^ | 10.9 (15.9) | 10.6 (17.6) | 0.3 (12.6) | 0.909 | 0.02 | -0.36 ; 0.32 |
| Systemic Chemotherapy Side Effects ^b^ | 16.7 (12.3) | 10.7 (9.8) | 6.0 (6.9) | < 0.001 | 0.49 | -0.87 ; -0.11 |
| Vaginal Symptoms ^b^ | 7.4 (12.8) | 7.4 (12.8) | 0.0 (0.0) | --- | --- | --- |
| Endocrine Symptoms ^b^ | 13.6 (16.6) | 11.9 (13.0) | 1.7 (7.2) | 0.266 | 0.10 | -0.45 ; 0.24 |
| Hand/foot symptoms/neuropathy ^b^ | 28.8 (20.0) | 24.0 (18.8) | 4.9 (19.0) | 0.223 | 0.25 | -0.60 ; 0.11 |
| Weight Gain ^b^ | 13.9 (16.8) | 13.9 (16.8) | 0.0 (9.8) | 1.000 | --- | --- |
| Skeletal ^b^ | 49.7 (29.9) | 35.1 (21.6) | 14.6 (24.2) | 0.007 | 0.49 | -0.87 ; -0.11 |

* Negative values indicate worsening.

a. Scores range from 0 to 100, with higher scores representing higher functional levels.

b. Scores range from 0 to 100, with higher scores representing greater degrees of symptoms.

# **Supplementary Table 8. Sensitivity to change according to the hypothesis of worsening in the global QOL item**

*Only patients whose global QOL item worsened between the first and second evaluations. ET cohort (n = 23)*

| **ET cohort** | First evaluation  Mean (SD) | Second evaluation  Mean (SD) | Change*  Mean (SD) | *p*-value | ES | 95% IC |
| --- | --- | --- | --- | --- | --- | --- |
| Body Image ^a^ | 87.7 (25.6) | 83.3 (25.7) | -4.3 (16.8) | 0.228 | -0.17 | -0.58 ; 0.25 |
| Sexual Function ^a^ | 4.8 (10.2) | 6.0 (15.5) | 1.2 (12.2) | 0.720 | 0.12 | -0.29 ; 0.53 |
| Sexual Enjoyment ^a^ | 16.7 (23.6) | 16.7 (23.6) | 0.0 (0.0) | --- | --- | --- |
| Future Perspective ^a^ | 76.8 (32.5) | 66.7 (26.6) | -10.1 (36.8) | 0.200 | -0.31 | -0.74 ; 0.12 |
| Breast Satisfaction ^a^ | 81.0 (17.8) | 61.9 (23.0) | -19.0 (32.5) | 0.172 | -1.07 | -1.67 ; -0.47 |
| Arm Symptoms ^b^ | 11.1 (16.4) | 6.3 (12.9) | 4.8 (8.1) | 0.009 | 0.29 | -0.72 ; 0.13 |
| Breast Symptoms ^b^ | 10.1 (11.8) | 10.5 (10.7) | -0.4 (10.2) | 0.866 | -0.03 | -0.38 ; 0.44 |
| Systemic Chemotherapy Side Effects ^b^ | 8.6 (7.7) | 16.5 (23.2) | -7.8 (22.9) | 0.440 | -1.01 | 0.43 ; 1.59 |
| Vaginal Symptoms ^b^ | --- | --- | --- | --- | --- | --- |
| Endocrine Symptoms ^b^ | 12.2 (26.8) | 13.3 (15.2) | -1.1 (21.3) | 0.903 | -0.04 | -0.37 ; 0.45 |
| Hand/feet symptoms/neuropathy ^b^ | 17.9 (18.9) | 32.1 (23.3) | -14.3 (36.6) | 0.341 | -0.76 | 0.24 ; 1.27 |
| Weight Gain ^b^ | 9.5 (25.2) | 14.3 (26.2) | -4.8 (40.5) | 0.766 | -0.19 | -0.23 ; 0.61 |
| Skeletal ^b^ | 28.6 (15.9) | 34.5 (21.7) | -6.0 (29.5) | 0.613 | -0.38 | -0.06 ; 0.81 |

* Negative values indicate worsening.

a. Scores range from 0 to 100, with higher scores representing higher functional levels.

b. Scores range from 0 to 100, with higher scores representing greater degrees of symptoms.
